# Supplementary material for: Topographic organization of eye-position dependent gain fields in human visual cortex
Source: Nat Commun. 2022 Dec 24;13:7925. doi: 10.1038/s41467-022-35488-8 (PMC9789150; doi:10.1038/s41467-022-35488-8)
Supplement: Supplementary file 1 — Supplementary Information [file 41467_2022_35488_MOESM1_ESM.pdf]

SUPPLEMENTARY INFORMATION

Topographic organization of eye-position dependent gain fields in human visual cortex

Jasper H. Fabius, Katarina Moravkova & Alessio Fracasso

Content

Supplementary Figures

- Supplementary Figure 1 p. 2
- Supplementary Figure 2 p. 3

- Supplementary Figure 3 p. 3
- Supplementary Figure 4 p. 5
- Supplementary Figure 5 p. 6
- Supplementary Figure 6 p. 7
- Supplementary Figure 7 p. 8
- Supplementary Figure 8 p. 9
- Supplementary Figure 9 p. 9
- Supplementary Figure 10 p. 10
- Supplementary Figure 11 p. 10

Supplementary Tables

All tables are part of a Supplementary Note.

- Supplementary Table 1 p. 4
- Supplementary Table 2 p. 11
- Supplementary Table 3 p. 12
- Supplementary Table 4 p. 14
- Supplementary Table 5 p. 15
- Supplementary Table 6 p. 19

Supplementary Notes

- Supplementary Note 1 p.11
  Linear mixed-effects model of change in R^2 between the pRF model not optimized for stimulus configuration and the pRF model optimized for stimulus configuration (pRF-only model), per ROI
- Supplementary Note 2 p.12
  Linear mixed-effects model of change in R^2_{adj} per ROI as a result of the addition of the pEGF to the pRF-only model
- Supplementary Note 3 p.14
  Reconstruction correlation coefficients and p-values per participant
- Supplementary Note 4 p.15
  Linear mixed-effects model of average correlation between actual and reconstructed eye position components per ROI
- Supplementary Note 5 p.17
  Linear mixed-effects model of pEGF X_0
- Supplementary Note 6 p.18
  Linear mixed-effects model of pEGF Y_0
- Supplementary Note 7 p.19
  PEGF center inversion

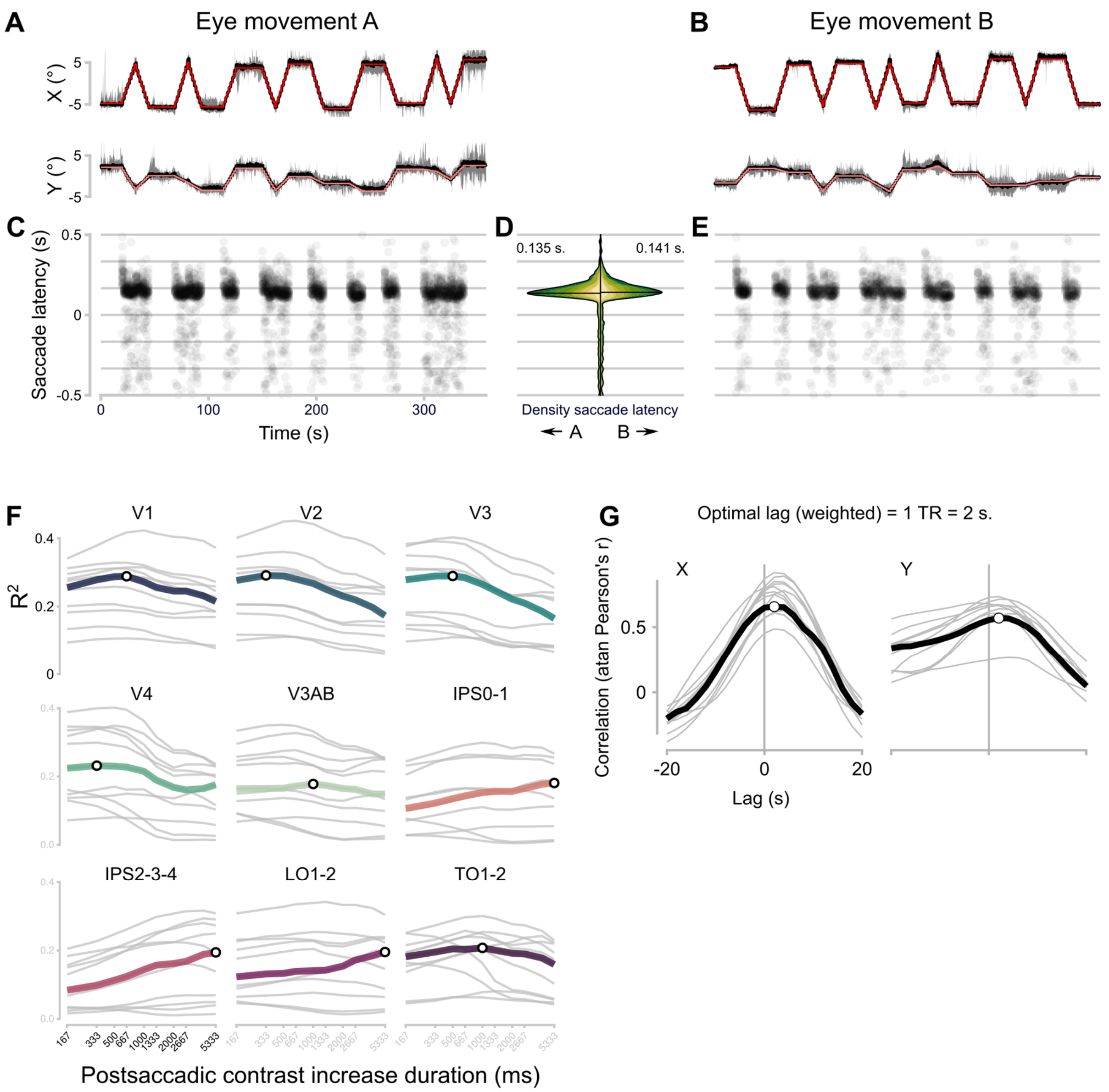

Supplementary Figure 1. A. Median horizontal (X) and vertical (Y) eye position components in eye movement task A. Black line is the median across participants. Shaded area represents the minimum and maximum across participants. Red and pink lines represent the actual target positions horizontal and vertical components, respectively. **B.** Like A, but for eye movement task B**. C.** Onset of detected saccades around the time of a change in position of the target. Each point represents a single saccade onset. **D.** Stacked density of the detected saccade onsets in C and E: left side represents the saccade latency in eye movement task A, right side represents latencies in eye movement task B. Colors from yellow to green represent different participants (in arbitrary order). Values represent the grand median across all detected saccades. **E.** Like C, but for saccades detected in eye movement task B. **F.** Median variance explained (R^2) using different stimulus models for eye movement task A. The difference between the models was in the duration of the postsaccadic contrast increase of the elements in the peripheral visual field. For this analysis, the contrast of the peripheral elements was set to 0% during periods of fixation and increased to 100% after a saccade. Grey lines represent the median R^2 in a different ROIs per participant. Colored lines represent the median across participants. Points represent the highest variance explained. In our analyses we used a duration of 500 ms, which best matched the optimal duration in early visual areas (V1, V2 and V3). **G.** Cross-correlation between reconstructed and actual eye position. Positive lags indicate the reconstruction is trailing the actual eye position. Correlation coefficients are Fisher transformed. Grey lines represent individual participants. The black line represents the group median. The white point indicates the maximum correlation per eye position component (x and y).

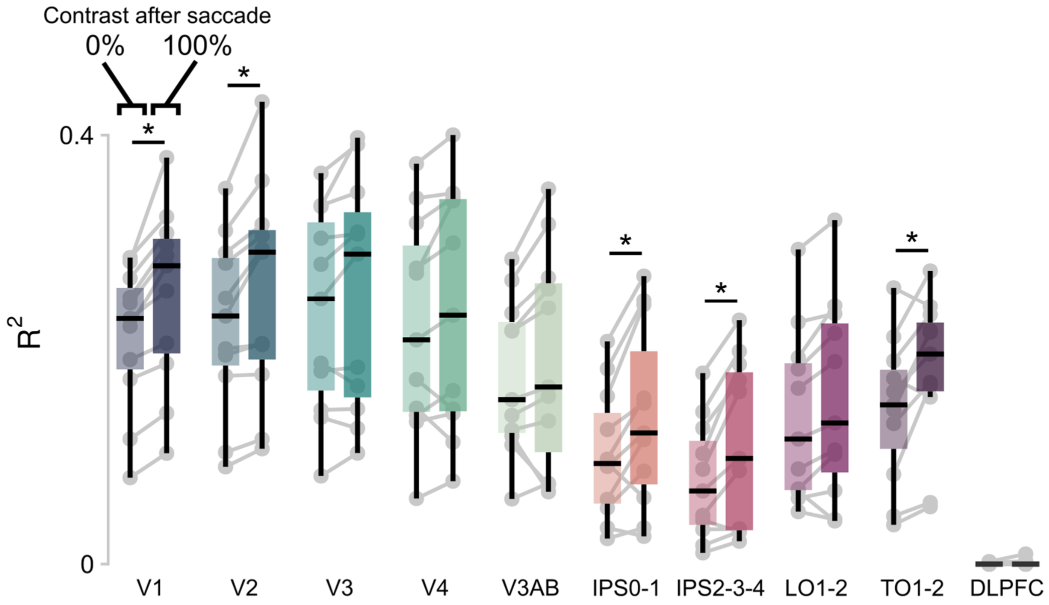

Supplementary Figure 2. Change in the variance explained (R^2) of BOLD responses in the eye-movement task by the pRF-only model (optimized for stimulus configuration, right boxes) compared to the pRF model not optimized for stimulus configuration (left boxes). For each pair of boxes, the left box represents the R^2 of the model that did not include the peripheral elements, the right box represents the R^2 of the model where the peripheral elements were modelled with full contrast for a period of 500 ms after saccade offset (pRF-only model). In the box plots, the center is the median, the box bounds are Q1 and Q3, the whiskers extend to the highest/lowest values with a max/min of 1.5× the IQR, data beyond these limits are shown as points. Grey lines and points represent single participants (N = 11). R^2 is computed as the median over all voxels of the most visually responsive voxels (i.e. the same as for Figure 3B). Asterisks indicate a significant difference (see all test results in Supplementary Information – Statistics Output – 1.). Overall, the pRF-only model yielded an increase in R^2 of approximately 0.035 in V1, V2, IPS0-1, IPS2-3-4 and TO1-2, compared to the pRF model not optimized for stimulus configuration.

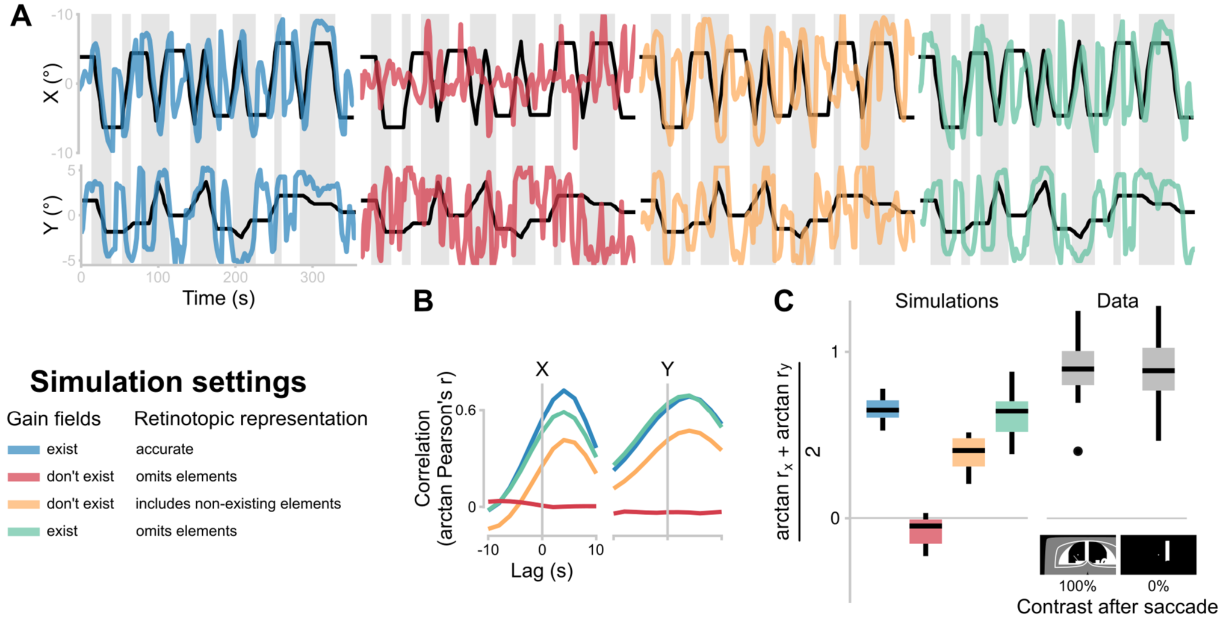

Supplementary Figure 3. Results of Simulation 1 (* see additional explanation below). **A.** Eye-position reconstruction results from the different simulation settings. **B.** Cross-correlation results. For three out of four simulations the maximum correlations are obtained for a lag of 2 TR, similar to the lag with maximum correlation for actual data. **C.** Average Fisher transformed correlation coordinates for the simulations and actual reconstructions (grey) with a lag of 1 TR. The boxplots capture the variability across participants (N = 11). Differences in reconstruction quality were assessed with a linear mixed-effects model: correlation ~ scenario + (1|participant), where correlation is the Fisher transformed average of the two components (X and Y). We bootstrapped the model estimates 10^6 times to obtain 95%-confidence intervals (corrected for multiple tests with Bonferroni's correction; see table below). In the box plot, the center is the median, the box bounds are Q1 and Q3, the whiskers extend to the highest/lowest values with a max/min of 1.5× the IQR, data beyond these limits are shown as points.

<u>*Additional explanation</u>
We considered four scenarios (see *Methods – Simulation 1: left-over retinotopic input* for details):

1) **Blue**: time-series were generated from both pRFs and pEGFs. The retinotopic representation of the visual input included the peripheral elements both when simulating the time-series and when estimating the pEGF parameters. This scenario follows the assumptions we make in our encoding model.
2) **Red**: time-series were generated from only the pRFs. The retinotopic representation included the peripheral elements when simulating the time-series, but not when estimating the pEGF parameters. This scenario captures reconstruction from left-over retinotopic activity.
3) **Yellow**: time-series were generated from only the pRFs. The retinotopic representation did not include the peripheral elements when simulating the time-series but did include them when estimating pEGF parameters. This scenario explores how well eye position can be reconstructed in case our stimulus model includes an excess of retinotopic elements.
4) **Green**: time-series were generated from both the pRFs and pEGFs. The retinotopic representation included the peripheral elements when simulating the time-series, but not when estimating the pEGF parameters. This scenario shows how well eye position can be reconstructed in case there is unaccounted retinotopic stimulation but gain fields do contribute to the time-series.

In all simulated scenarios except scenario 2 (red), eye position reconstruction was above chance level. In scenario 2 (red), reconstruction was at chance level, indicating that accurate reconstruction from only left-over retinotopic activity is unlikely. However, scenario 3 (yellow) also leads to above chance level reconstruction, even though there were no pEGFs simulated. It is thus possible that our reconstruction did not rely on the existence of pEGFs but on a faulty retinotopic representation, that included an excess of peripheral elements. Still, reconstruction quality in the yellow scenario was significantly lower than in the blue scenario, which captures our two assumptions: that the peripheral elements are part of the visual input and that the gain of visual responses is modulated by eye position. In addition, results from the green scenario show that as long as pEGFs are real, omitting the peripheral elements should not hamper the reconstruction of eye position; there is no significant difference between the blue and green scenarios. To test whether our reconstruction results from the actual data might arise from a faulty stimulus model that includes too many peripheral elements (like is the yellow scenario), we re-estimated pEGF parameters using a retinotopic representation wherein the contrast of the peripheral elements was kept at 0 all the time. If the reconstruction quality would drop to chance level, we would know our data are unlikely to be driven by pEGFs, because the drop to chance level would be similar to the difference between the yellow and red scenario. However, if reconstruction quality would stay the same, it is more likely that our data are driven by pEGFs (like the blue and green scenarios). In panel C, the reconstruction quality for both versions of the retinotopic representation is displayed. Reconstruction quality is not significantly different between the two representations ($F(1,10) = 1.61$, $p = 0.23$).Together, the results from this simulation and the results from the real data in panel C indicate that the observed reconstruction most likely results from pEGFs truly affecting the measured BOLD time-series.

Supplementary Table 1

Scenario A	Scenario B	Median	2.5%	97.5%	Significant
Blue (1)	-	0.6877	0.5945	0.7804	TRUE
Red (2)	-	-0.0851	-0.1785	0.0089	FALSE
Yellow (3)	-	0.4213	0.3289	0.5148	TRUE
Green (4)	-	0.6300	0.5371	0.7229	TRUE
Blue (1)	Red (2)	0.7731	0.6862	0.8591	TRUE
Blue (1)	Yellow (3)	0.2664	0.1803	0.3514	TRUE
Blue (1)	Green (4)	0.0577	-0.0288	0.1438	FALSE
Red (2)	Yellow (3)	-0.5064	-0.5927	-0.4204	TRUE
Red (2)	Green (4)	-0.7152	-0.8003	-0.6299	TRUE
Yellow (3)	Green (4)	-0.2088	-0.2944	-0.1235	TRUE

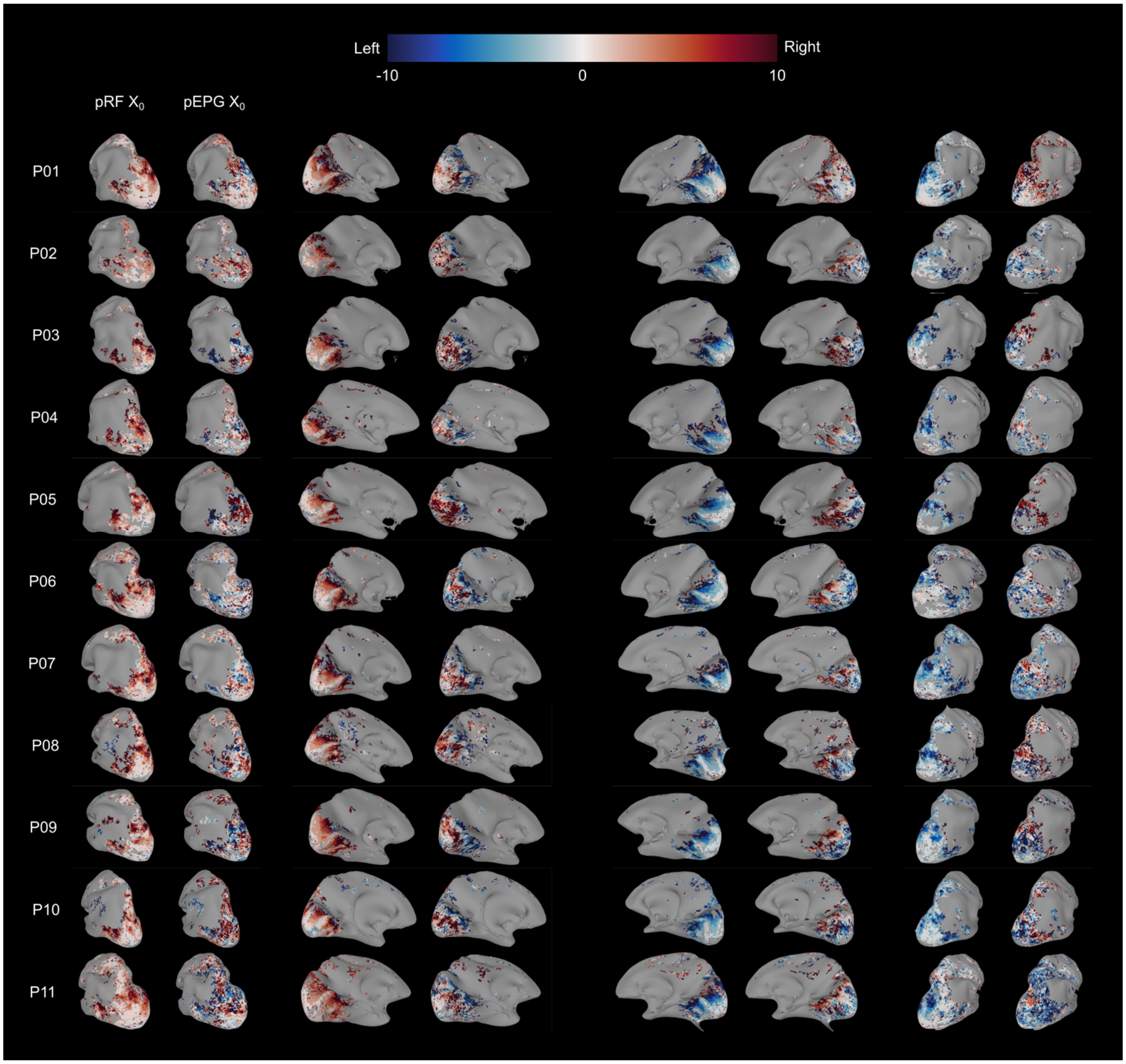

Supplementary Figure 4. Surface maps of horizontal components (X_0) of pRF and pEGF centers for all participants. Each row is a participant, showing the left and right, lateral and medial maps of pRF and pEGF X_0. The pEGF X_0 shows a gradient from contralateral to ipsilateral along the posterior-anterior axis, the same direction as eccentricity of the pRFs. Maps are thresholded based on the R^2 of the pRF×pEGF model. The minimum R^2 was set to 0.1 (i.e. at least 10% variance explained).

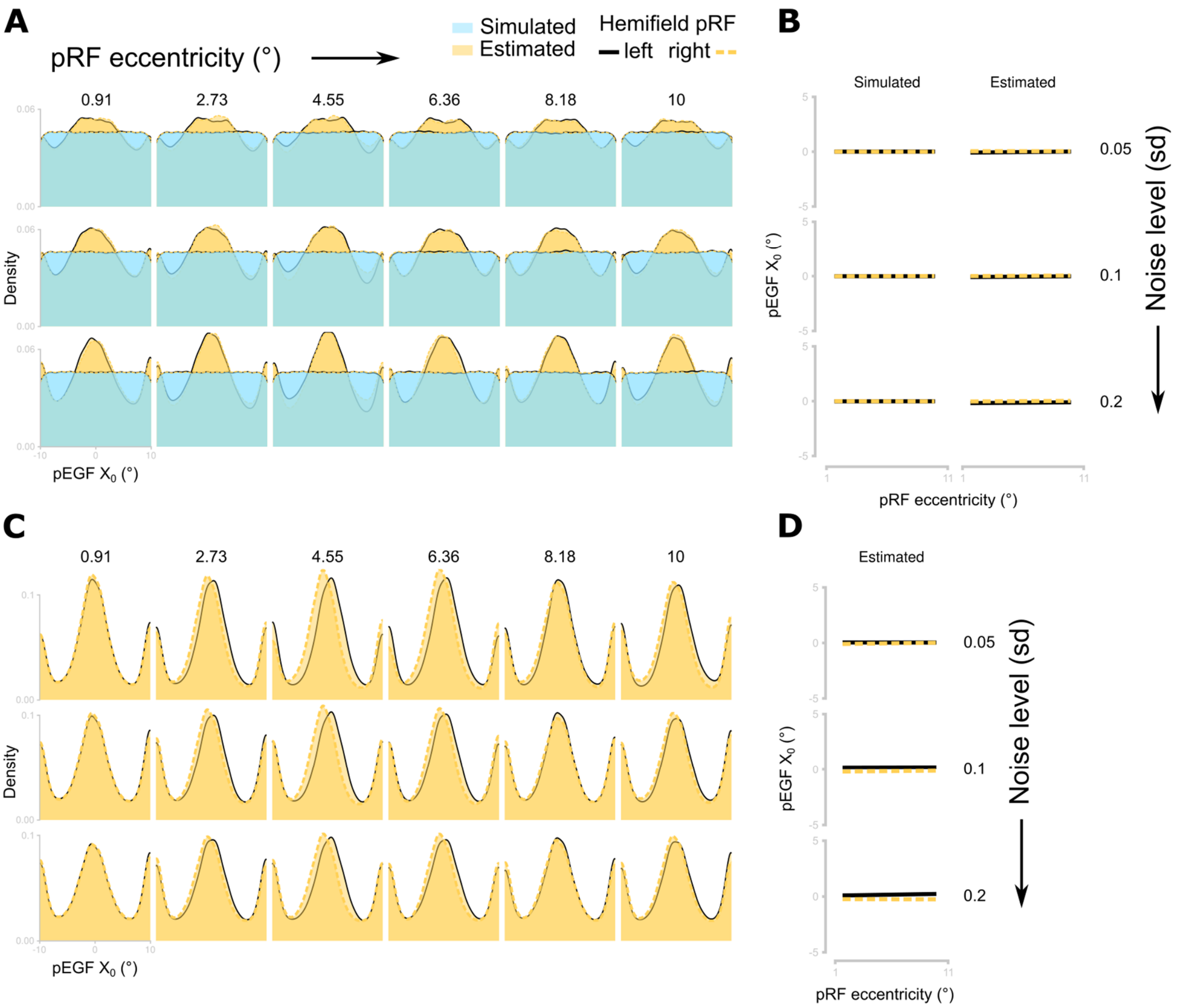

Supplementary Figure 5. Results of Simulation 2. **A.** Simulated (blue) and estimated (yellow) horizontal component (X_0) of pEGF centers. The difference between the simulated and estimated pEGF X_0 is due to the structure of our modelling framework. **B.** Average pEGF X_0 per over pRF eccentricity. The relationship between pRF eccentricity and pEGF X_0 does not show the inversion that is observed in the data. **C.** Like A, but in this simulation the pEGF was omitted entirely. **D.** Like B. Also, when the pEGFs are omitted entirely from the simulation, the inversion of pEGF center with pRF eccentricity does not emerge.

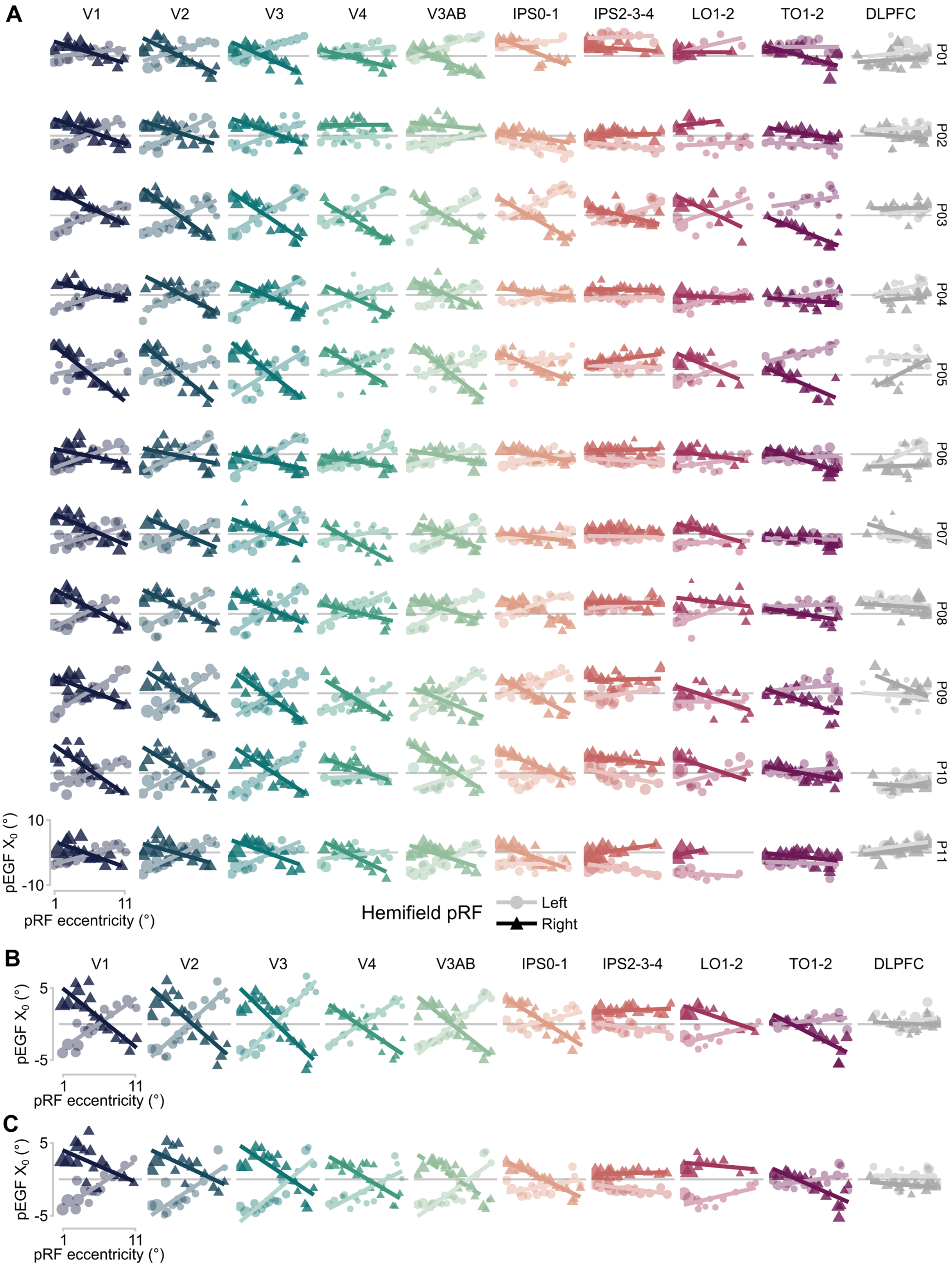

Supplementary Figure 6. A. Relationship between pRF eccentricity and the horizontal component (X_0) of the pEGF per participant (rows) and visual area (columns). **B.** Group average, same plots as Figure 5D and 5E. To estimate the pEGF X_0 estimates we used a configuration of the retinotopic representation of the visual input where the contrast of the peripheral elements increased to 100% after each saccade. **C.** Like B, but here the pEGF X_0 estimates were obtained with a version of the retinotopic representation of the visual input where the contrast of the peripheral elements was kept at 0% at all times. I.e., in this representation, only the flickering bars and fixation point provided a visual input.

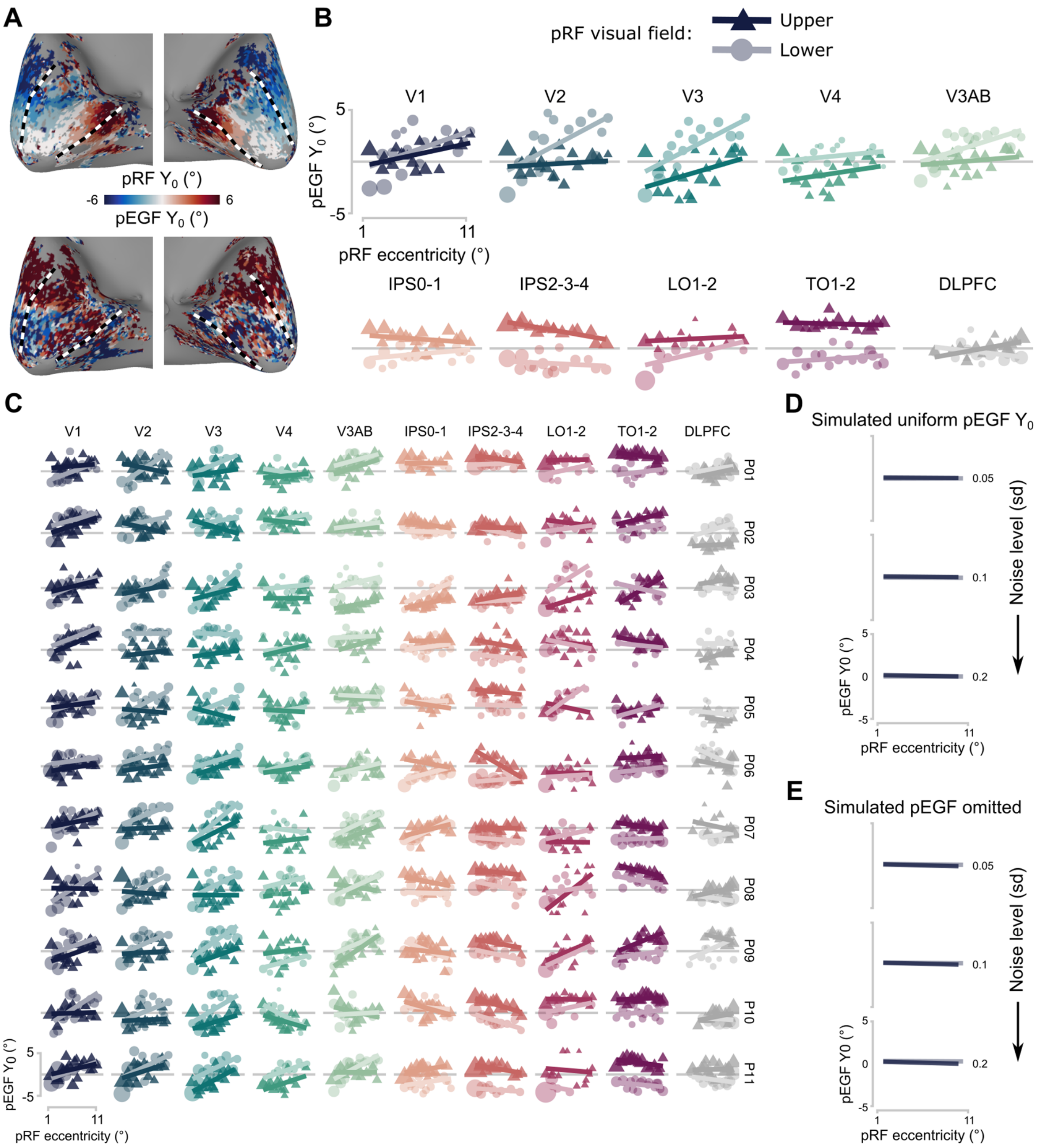

Supplementary Figure 7. Topographic organization of the vertical component (Y_0) of pEGF centers. This organization is less pronounced than for the horizontal component (X_0) and is more heterogeneous across visual ROIs. **A.** Example surface maps with the vertical component of pRFs (top) and pEGF (bottom). Dashed lines mark the borders between V1 and V2. **B.** Average relationship between pRF eccentricity and pEGF Y_0. Data are split between pRFs in the upper and lower visual field. **C.** Similar plots like in panel B for individual participants and ROIs. **D.** Results of simulation 2: recovered pEGF Y_0 after simulating a uniform distribution of pEGF Y_0. Different panels correspond to different noise levels. **E.** Results of simulation 2: recovered pEGF Y_0 after omitting the pEGF from the simulated time series.

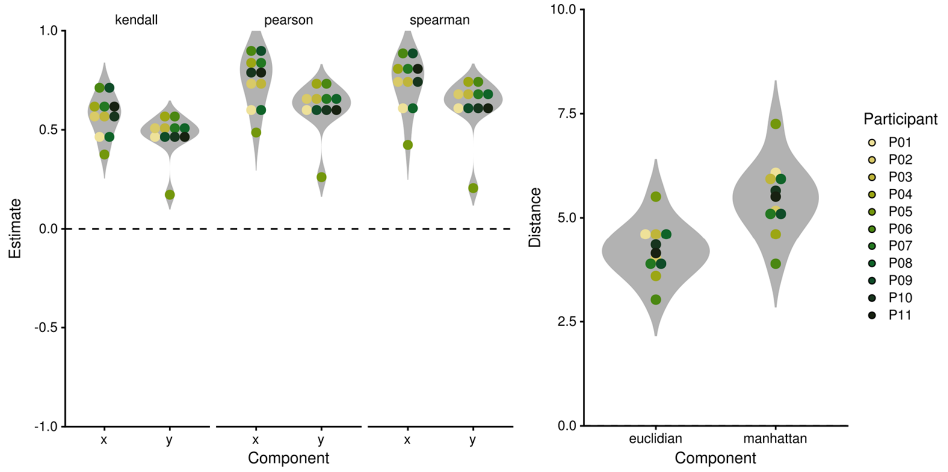

Supplementary Figure 8. Alternative correlation and distance metrics. NB: These figures have been used in rebuttals to the reviewers but have not been used further in the manuscript.

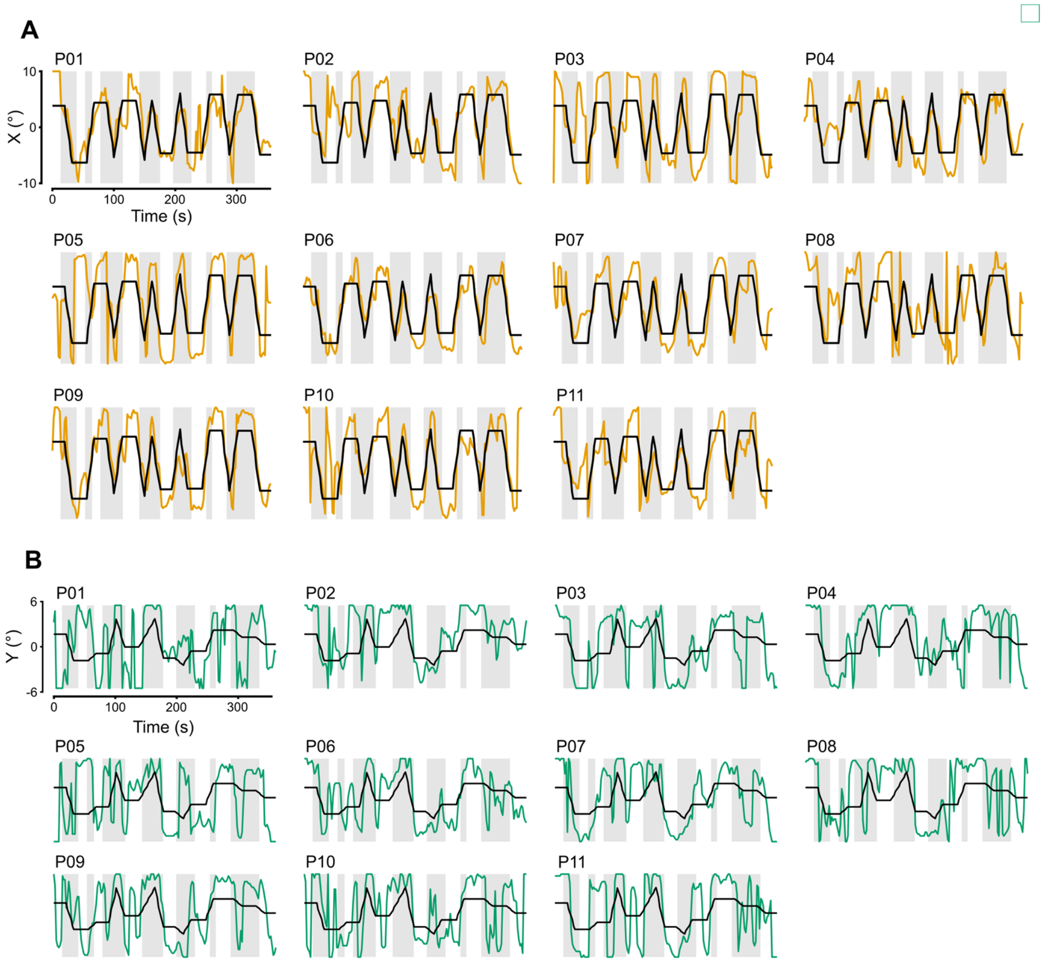

Supplementary Figure 9. Individual eye position estimates per participant. **A.** Horizontal eye position **B.** Vertical eye position. NB: These figures have been used in rebuttals to the reviewers but have not been used further in the manuscript.

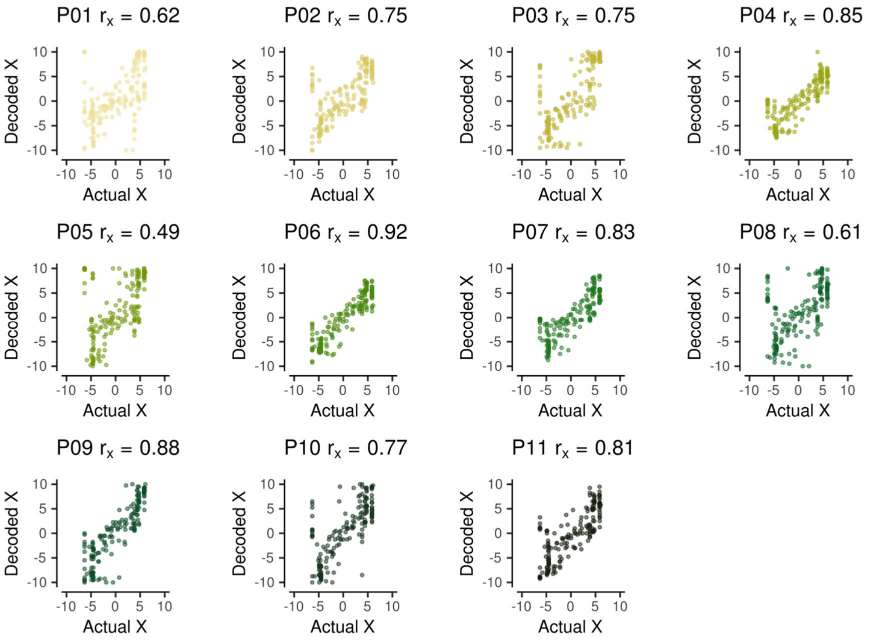

Supplementary Figure 10. Individual scatter plots between estimated and actual horizontal eye positions per participant. NB: These figures have been used in rebuttals to the reviewers but have not been used further in the manuscript.

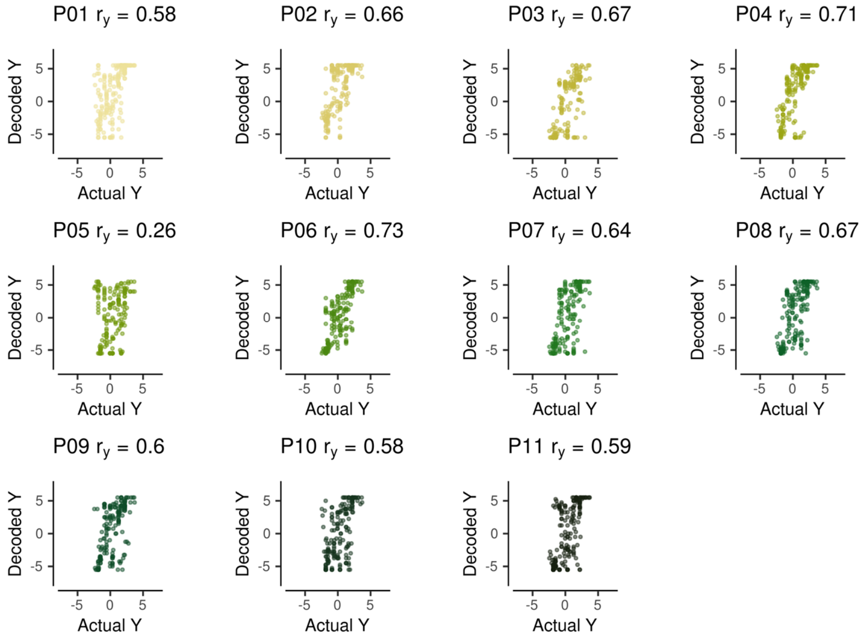

Supplementary Figure 11. Individual scatter plots between estimated and actual vertical eye positions per participant. NB: These figures have been used in rebuttals to the reviewers but have not been used further in the manuscript.

Supplementary notes

Supplementary Note 1. Linear mixed-effects model of change in R2 between the pRF model not optimized for stimulus configuration and the pRF model optimized for stimulus configuration (pRF-only model), per ROI.

```
Command:
anova(mdl0, mdl1)

Models:
mdl0: dR2 ~ 0 + (1 | subj)
mdl1: dR2 ~ roi + (1 | subj)

     npar     AIC     BIC logLik deviance  Chisq Df Pr(>Chisq)
mdl0    2 -510.11 -504.71 257.06  -514.11
mdl1   12 -552.25 -519.85 288.13  -576.25 62.14 10  1.423e-09 ***

Command:
anova(mdl1)

Type III Analysis of Variance Table with Satterthwaite's method
      Sum Sq   Mean Sq NumDF DenDF F value    Pr(>F)
roi 0.016269 0.0018077     9    90  7.1565 9.188e-08 ***
```

Confidence intervals of the changes in R^2 per ROI. Estimates are derived by bootstrapping mdl1 100000 times using the “bootMer” and “confint” functions from the “boot” package in R. Estimates are corrected for multiple comparisons using Bonferroni’s correction, by adjusting the limits of the confidence interval by the number of comparisons (= 10). I.e. the limits of a two-sided 95%-confidence interval become the (2.5/10=) 0.25th percentile and (100-2.5/10) = 99.75th percentile. Significance is determined by whether or not 0 is included in the confidence interval.

Supplementary Table 2

ROI	median	q025	q975	q025 *corrected*	q975 *corrected*	significant†
V1	0.0391	0.0230	0.0550	0.0162	0.0630	*
V2	0.0300	0.0139	0.0460	0.0068	0.0530	*
V3	0.0158	−0.0003	0.0320	−0.0072	0.0390	
V4	0.0142	−0.0019	0.0300	−0.0089	0.0370	
V3AB	0.0159	−0.0001	0.0320	−0.0071	0.0390	
IPS0-1	0.0348	0.0188	0.0510	0.0120	0.0580	*
IPS2-3-4	0.0355	0.0194	0.0520	0.0126	0.0590	*
LO1/LO2	0.0163	0.0002	0.0320	−0.0064	0.0390	°
TO1/TO2	0.0360	0.0200	0.0520	0.0129	0.0590	*
DLPFC	0.0009	−0.0151	0.0170	−0.0220	0.0240	

†*Significance classification:*
** = 0 outside Bonferroni-corrected 95%-confidence interval*
° = 0 outside uncorrected 95%-confidence interval

Supplementary Note 2. Linear mixed-effects model of change in R^2_{adj} per ROI as a result of the addition of the pEGF to the pRF-only model

```
Command:
anova(mdl0, mdl1)

Models:
mdl0: dR2adj ~ 0 + (1 | subj)
mdl1: dR2adj ~ roi + (1 | subj)

     npar     AIC     BIC logLik deviance  Chisq Df Pr(>Chisq)
mdl0    2 -517.20 -511.79 260.60  -521.20
mdl1   12 -673.05 -640.64 348.52  -697.05 175.85 10  < 2.2e-16 ***
```

```
Command:
anova(mdl1)

Type III Analysis of Variance Table with Satterthwaite's method
      Sum Sq   Mean Sq NumDF DenDF F value    Pr(>F)
roi 0.026515 0.0029461     9    90   30.77 < 2.2e-16 ***
```

Confidence intervals of the changes in $R2_{adj}$ per ROI and differences in changes between ROIs. Estimates are derived by bootstrapping mdl1 100000 times.

Supplementary Table 3

ROI 1	ROI 2	median	q025	q975	q025 *corrected*	q975 *corrected*	significant†
V1	-	0.0593	0.0523	0.0664	0.0474	0.0713	*
V2	-	0.0526	0.0456	0.0596	0.0407	0.0643	*
V3	-	0.0545	0.0476	0.0615	0.0426	0.0665	*
V4	-	0.0344	0.0274	0.0414	0.0226	0.0463	*
V3AB	-	0.0393	0.0323	0.0462	0.0272	0.0512	*
IPS0-1	-	0.0271	0.0200	0.0341	0.0153	0.0386	*
IPS2-3-4	-	0.0251	0.0181	0.0322	0.0135	0.0369	*
LO1/LO2	-	0.0370	0.0300	0.0440	0.0250	0.0489	*
TO1/TO2	-	0.0348	0.0278	0.0418	0.0229	0.0468	*
DLPFC	-	0.0037	-0.0033	0.0107	-0.0081	0.0155	
V1	V2	0.0068	-0.0014	0.0150	-0.0072	0.0204	
V1	V3	0.0048	-0.0033	0.0130	-0.0092	0.0189	
V1	V4	0.0249	0.0167	0.0331	0.0110	0.0388	*
V1	V3AB	0.0201	0.0119	0.0283	0.0064	0.0339	*
V1	IPS0-1	0.0323	0.0240	0.0404	0.0186	0.0463	*
V1	IPS2-3-4	0.0342	0.0260	0.0424	0.0204	0.0480	*
V1	LO1/LO2	0.0223	0.0141	0.0305	0.0086	0.0361	*
V1	TO1/TO2	0.0245	0.0163	0.0327	0.0103	0.0384	*
V1	DLPFC	0.0556	0.0474	0.0638	0.0419	0.0697	*
V2	V3	-0.0020	-0.0101	0.0062	-0.0157	0.0120	
V2	V4	0.0182	0.0100	0.0264	0.0043	0.0320	*
V2	V3AB	0.0133	0.0051	0.0215	-0.0007	0.0270	°
V2	IPS0-1	0.0255	0.0173	0.0337	0.0117	0.0393	*
V2	IPS2-3-4	0.0274	0.0192	0.0356	0.0133	0.0414	*
V2	LO1/LO2	0.0155	0.0074	0.0237	0.0017	0.0295	*
V2	TO1/TO2	0.0178	0.0096	0.0259	0.0037	0.0315	*
V2	DLPFC	0.0488	0.0406	0.0571	0.0350	0.0624	*
V3	V4	0.0201	0.0120	0.0283	0.0065	0.0341	*
V3	V3AB	0.0153	0.0071	0.0234	0.0014	0.0293	*
V3	IPS0-1	0.0274	0.0193	0.0357	0.0137	0.0413	*
V3	IPS2-3-4	0.0294	0.0212	0.0375	0.0157	0.0431	*
V3	LO1/LO2	0.0175	0.0093	0.0257	0.0038	0.0314	*
V3	TO1/TO2	0.0197	0.0115	0.0279	0.0059	0.0336	*
V3	DLPFC	0.0508	0.0427	0.0590	0.0370	0.0648	*
V4	V3AB	-0.0049	-0.0130	0.0033	-0.0187	0.0089	
V4	IPS0-1	0.0073	-0.0009	0.0155	-0.0066	0.0210	
V4	IPS2-3-4	0.0093	0.0010	0.0174	-0.0047	0.0232	°
V4	LO1/LO2	-0.0026	-0.0108	0.0055	-0.0164	0.0111	
V4	TO1/TO2	-0.0004	-0.0086	0.0077	-0.0141	0.0135	
V4	DLPFC	0.0307	0.0226	0.0389	0.0168	0.0444	*
V3AB	IPS0-1	0.0122	0.0040	0.0204	-0.0017	0.0263	°
V3AB	IPS2-3-4	0.0141	0.0060	0.0223	0.0002	0.0280	*
V3AB	LO1/LO2	0.0022	-0.0060	0.0104	-0.0115	0.0163	

V3AB	TO1/TO2	0.0045	−0.0037	0.0127	−0.0094	0.0186	
V3AB	DLPFC	0.0355	0.0274	0.0437	0.0216	0.0493	*
IPS0-1	IPS2-3-4	0.0019	−0.0062	0.0101	−0.0121	0.0159	
IPS0-1	LO1/LO2	−0.0100	−0.0181	−0.0018	−0.0237	0.0043	°
IPS0-1	TO1/TO2	−0.0077	−0.0160	0.0004	−0.0218	0.0061	
IPS0-1	DLPFC	0.0234	0.0152	0.0315	0.0093	0.0369	*
IPS2-3-4	LO1/LO2	−0.0119	−0.0200	−0.0037	−0.0258	0.0019	°
IPS2-3-4	TO1/TO2	−0.0097	−0.0178	−0.0015	−0.0237	0.0043	°
IPS2-3-4	DLPFC	0.0214	0.0133	0.0296	0.0077	0.0352	*
LO1/LO2	TO1/TO2	0.0022	−0.0059	0.0104	−0.0116	0.0159	
LO1/LO2	DLPFC	0.0333	0.0252	0.0415	0.0195	0.0474	*
TO1/TO2	DLPFC	0.0311	0.0229	0.0393	0.0173	0.0452	*

†*Significance classification:*

** = 0 outside Bonferroni-corrected 95%-confidence interval*

° = 0 outside uncorrected 95%-confidence interval

Supplementary Note 3. Reconstruction correlation coefficients and p-values per participant

Correlation coefficients and p-values of reconstruction correlations compared to a null distribution of correlation coefficients obtained by reconstructing after permuting pEGF parameters

Supplementary Table 4

Participant	Pearson's r_x	p value	Pearson's r_y	p value
P01	0.617	0.000	0.582	0.000
P02	0.749	0.000	0.664	0.000
P03	0.752	0.000	0.672	0.000
P04	0.847	0.000	0.712	0.000
P05	0.486	0.005	0.261	0.084
P06	0.915	0.000	0.732	0.000
P07	0.828	0.000	0.639	0.000
P08	0.607	0.000	0.666	0.000
P09	0.881	0.000	0.604	0.000
P10	0.770	0.000	0.585	0.000
P11	0.807	0.000	0.595	0.000

Supplementary Note 4. Linear mixed-effects model of average correlation between actual and reconstructed eye position components per ROI

Linear mixed-effects model of the average Fisher transformed Pearson correlations coefficients of the two reconstructed eye position components with the actual eye position per ROI.

```
Command:
anova(mdl0, mdl1)

Models:
mdl0: estimate ~ 1 + (1 | subj)
mdl1: estimate ~ roi + (1 | subj)
     npar     AIC     BIC  logLik deviance  Chisq Df Pr(>Chisq)
mdl0    3  50.282  58.384 -22.141   44.282
mdl1   12 -87.079 -54.673  55.540 -111.079 155.36  9  <2.2e-16 ***
```

```
Command:
anova(mdl1)

Type III Analysis of Variance Table with Satterthwaite's method
    Sum Sq Mean Sq NumDF DenDF F value  Pr(>F)
roi 6.3391 0.70435     9    90  38.033  <2.2e-16 ***
```

Confidence intervals of average Fisher transformed Pearson correlations per ROI and differences in changes between ROIs. Estimates are derived by bootstrapping mdl1 100,000 times.

Supplementary Table 5

ROI 1	ROI 2	median	q025	q975	q025 *corrected*	q975 *corrected*	significant†
V1	-	0.791	0.678	0.903	0.601	0.981	*
V2	-	0.863	0.750	0.975	0.675	1.053	*
V3	-	0.814	0.702	0.927	0.622	1.006	*
V4	-	0.465	0.353	0.578	0.276	0.656	*
V3AB	-	0.706	0.594	0.819	0.519	0.898	*
IPS0-1	-	0.555	0.442	0.667	0.366	0.743	*
IPS2-3-4	-	0.359	0.247	0.472	0.173	0.550	*
LO1/2	-	0.385	0.272	0.497	0.194	0.579	*
TO1/2	-	0.309	0.197	0.422	0.119	0.500	*
DLPFC	-	0.103	-0.010	0.215	-0.088	0.298	
V1	V2	-0.072	-0.186	0.042	-0.266	0.124	
V1	V3	-0.024	-0.137	0.091	-0.218	0.170	
V1	V4	0.326	0.211	0.440	0.132	0.517	*
V1	V3AB	0.084	-0.029	0.198	-0.111	0.271	
V1	IPS0-1	0.236	0.122	0.350	0.042	0.430	*
V1	IPS2-3-4	0.431	0.318	0.545	0.241	0.624	*
V1	LO1/2	0.406	0.293	0.520	0.213	0.602	*
V1	TO1/2	0.481	0.367	0.595	0.288	0.673	*
V1	DLPFC	0.688	0.575	0.802	0.495	0.885	*
V2	V3	0.048	-0.065	0.162	-0.141	0.240	
V2	V4	0.398	0.284	0.512	0.208	0.592	*
V2	V3AB	0.156	0.043	0.270	-0.036	0.350	°
V2	IPS0-1	0.308	0.195	0.421	0.117	0.501	*
V2	IPS2-3-4	0.503	0.390	0.617	0.313	0.694	*
V2	LO1/2	0.478	0.364	0.592	0.283	0.672	*
V2	TO1/2	0.553	0.440	0.667	0.361	0.746	*
V2	DLPFC	0.760	0.646	0.873	0.565	0.949	*
V3	V4	0.349	0.236	0.463	0.158	0.547	*
V3	V3AB	0.108	-0.006	0.222	-0.086	0.300	
V3	IPS0-1	0.260	0.145	0.374	0.070	0.456	*
V3	IPS2-3-4	0.455	0.341	0.569	0.263	0.643	*
V3	LO1/2	0.430	0.316	0.544	0.238	0.621	*
V3	TO1/2	0.505	0.391	0.618	0.310	0.702	*
V3	DLPFC	0.712	0.598	0.826	0.517	0.901	*
V4	V3AB	-0.241	-0.355	-0.127	-0.435	-0.051	*
V4	IPS0-1	-0.090	-0.203	0.024	-0.280	0.102	
V4	IPS2-3-4	0.106	-0.009	0.219	-0.086	0.295	
V4	LO1/2	0.080	-0.034	0.194	-0.113	0.274	
V4	TO1/2	0.156	0.042	0.269	-0.039	0.349	°
V4	DLPFC	0.362	0.248	0.477	0.167	0.551	*

V3AB	IPS0-1	0.152	0.037	0.266	-0.044	0.346	°
V3AB	IPS2-3-4	0.347	0.233	0.461	0.153	0.535	*
V3AB	LO1/2	0.322	0.208	0.436	0.130	0.514	*
V3AB	TO1/2	0.397	0.283	0.510	0.204	0.591	*
V3AB	DLPFC	0.604	0.490	0.718	0.408	0.798	*
IPS0-1	IPS2-3-4	0.195	0.081	0.310	0.004	0.391	*
IPS0-1	LO1/2	0.170	0.056	0.284	-0.022	0.363	°
IPS0-1	TO1/2	0.245	0.132	0.359	0.053	0.436	*
IPS0-1	DLPFC	0.452	0.339	0.566	0.259	0.649	*
IPS2-3-4	LO1/2	-0.025	-0.139	0.089	-0.219	0.171	
IPS2-3-4	TO1/2	0.050	-0.064	0.163	-0.140	0.239	
IPS2-3-4	DLPFC	0.257	0.142	0.371	0.062	0.456	*
LO1/2	TO1/2	0.076	-0.039	0.189	-0.119	0.270	
LO1/2	DLPFC	0.282	0.168	0.396	0.089	0.472	*
TO1/2	DLPFC	0.207	0.093	0.320	0.013	0.398	*

†Significance classification:

** = 0 outside Bonferroni-corrected 95%-confidence interval*

° = 0 outside uncorrected 95%-confidence interval

Supplementary Note 5. Linear mixed-effects model of pEGF X_0

Separate models were created for each ROI, to keep the factors in the model interpretable. For each ROI the model had the structure:

```
mdl <- lmer(pEGF X0 ~ prf.eccentricity * hemifield + (1 | subj), data)
Command:
anova(mdl)
Type III Analysis of Variance Table with Satterthwaite's method

V1
                              Sum Sq Mean Sq NumDF  DenDF  F value    Pr(>F)
prf.eccentricity               32.33   32.33     1 355.77   7.1254  0.007948 **
hemifield                    2157.71 2157.71     1 355.15 475.5539 < 2.2e-16 ***
prf.eccentricity:hemifield 2037.41 2037.41     1 355.77 449.0392 < 2.2e-16 ***

V2
                             Sum Sq Mean Sq NumDF  DenDF  F value  Pr(>F)
prf.eccentricity               15.6    15.6     1 354.90   2.8881 0.09011 .
hemifield                    2802.5  2802.5     1 353.00 519.9613 < 2e-16 ***
prf.eccentricity:hemifield 3250.4  3250.4     1 353.16 603.0689 < 2e-16 ***

V3
                             Sum Sq Mean Sq NumDF  DenDF  F value Pr(>F)
prf.eccentricity                8.0     8.0     1 361.72   1.5525 0.2136
hemifield                    3203.4  3203.4     1 359.84 621.7351 <2e-16 ***
prf.eccentricity:hemifield 4072.8  4072.8     1 360.16 790.4595 <2e-16 ***

V4
                              Sum Sq Mean Sq NumDF  DenDF  F value  Pr(>F)
prf.eccentricity               34.12   34.12     1 345.31   6.1892 0.01332 *
hemifield                     943.06  943.06     1 344.68 171.0536 < 2e-16 ***
prf.eccentricity:hemifield 1467.19 1467.19     1 344.87 266.1200 < 2e-16 ***

V3AB
                              Sum Sq Mean Sq NumDF  DenDF  F value Pr(>F)
prf.eccentricity                6.38    6.38     1 366.44   1.1325 0.2879
hemifield                    1957.15 1957.15     1 365.12 347.4613 <2e-16 ***
prf.eccentricity:hemifield 2449.75 2449.75     1 365.49 434.9147 <2e-16 ***

IPS0-1
                             Sum Sq Mean Sq NumDF  DenDF F value    Pr(>F)
prf.eccentricity             138.58  138.58     1 359.52  25.107 8.526e-07 ***
hemifield                    601.48  601.48     1 358.11 108.973 < 2.2e-16 ***
prf.eccentricity:hemifield 860.03  860.03     1 358.16 155.816 < 2.2e-16 ***

IPS2-3-4
                              Sum Sq Mean Sq NumDF  DenDF F value    Pr(>F)
prf.eccentricity               1.478   1.478     1 321.39  0.4079    0.5235
hemifield                    146.672 146.672     1 321.03 40.4929 6.802e-10 ***
prf.eccentricity:hemifield   5.293   5.293     1 321.08  1.4613    0.2276

LO1-2
                             Sum Sq Mean Sq NumDF  DenDF  F value    Pr(>F)
prf.eccentricity               8.16    8.16     1 216.52   1.2601    0.2629
hemifield                    683.30  683.30     1 215.70 105.5093 < 2.2e-16 ***
prf.eccentricity:hemifield 327.41  327.41     1 217.09  50.5555 1.652e-11 ***

TO1-2
                             Sum Sq Mean Sq NumDF  DenDF F value    Pr(>F)
prf.eccentricity              92.99   92.99     1 367.03 13.3171 0.0003011 ***
hemifield                     40.03   40.03     1 366.12  5.7324 0.0171572 *
prf.eccentricity:hemifield 335.24  335.24     1 366.19 48.0076  1.92e-11 ***

DLPFC
                             Sum Sq Mean Sq NumDF  DenDF F value Pr(>F)
prf.eccentricity             5.2299  5.2299     1 268.77  0.7956 0.3732
hemifield                    1.5384  1.5384     1 268.50  0.2340 0.6289
prf.eccentricity:hemifield 5.5463  5.5463     1 268.40  0.8438 0.3591
```

Supplementary Note 6. Linear mixed-effects model of pEGF Y_0

Separate models were created for each ROI, to keep the factors in the model interpretable. Hemifield here was ‘upper’ and ‘lower’. For each ROI the model had the structure:

V1

	Sum Sq	Mean Sq	NumDF	DenDF	F value	Pr(>F)	
prf.eccentricity	295.471	295.471	1	330.18	81.7807	< 2.2e-16	***
hemifield	9.320	9.320	1	329.53	2.5796	0.109208	
prf.eccentricity:hemifield	30.805	30.805	1	329.73	8.5261	0.003742	**

V2

	Sum Sq	Mean Sq	NumDF	DenDF	F value	Pr(>F)	
prf.eccentricity	156.176	156.176	1	326.77	38.973	1.334e-09	***
hemifield	13.669	13.669	1	326.35	3.411	0.06567	.
prf.eccentricity:hemifield	131.744	131.744	1	326.43	32.876	2.238e-08	***

V3

	Sum Sq	Mean Sq	NumDF	DenDF	F value	Pr(>F)	
prf.eccentricity	320.00	320.00	1	330.33	76.7419	< 2.2e-16	***
hemifield	3.34	3.34	1	330.01	0.8002	0.3717002	
prf.eccentricity:hemifield	60.14	60.14	1	329.98	14.4234	0.0001737	***

V4

	Sum Sq	Mean Sq	NumDF	DenDF	F value	Pr(>F)	
prf.eccentricity	17.4546	17.4546	1	313.24	3.9322	0.04824	*
hemifield	11.8673	11.8673	1	312.62	2.6735	0.10304	
prf.eccentricity:hemifield	0.7166	0.7166	1	312.63	0.1614	0.68811	

V3AB

	Sum Sq	Mean Sq	NumDF	DenDF	F value	Pr(>F)	
prf.eccentricity	143.321	143.321	1	338.66	40.9250	5.259e-10	***
hemifield	3.889	3.889	1	338.68	1.1106	0.29271	
prf.eccentricity:hemifield	11.052	11.052	1	338.92	3.1559	0.07655	.

IPS0-1

	Sum Sq	Mean Sq	NumDF	DenDF	F value	Pr(>F)	
prf.eccentricity	1.760	1.760	1	324.93	0.6002	0.4390574	
hemifield	39.639	39.639	1	324.38	13.5161	0.0002767	***
prf.eccentricity:hemifield	19.406	19.406	1	324.45	6.6170	0.0105455	*

IPS2-3-4

	Sum Sq	Mean Sq	NumDF	DenDF	F value	Pr(>F)	
prf.eccentricity	41.719	41.719	1	305.22	23.0035	2.532e-06	***
hemifield	136.665	136.665	1	305.06	75.3560	2.409e-16	***
prf.eccentricity:hemifield	3.887	3.887	1	305.11	2.1434	0.1442	

LO1-2

	Sum Sq	Mean Sq	NumDF	DenDF	F value	Pr(>F)	
prf.eccentricity	89.299	89.299	1	239.96	15.1984	0.0001256	***
hemifield	7.297	7.297	1	239.62	1.2418	0.2662336	
prf.eccentricity:hemifield	9.543	9.543	1	240.06	1.6242	0.2037415	

TO1-2

	Sum Sq	Mean Sq	NumDF	DenDF	F value	Pr(>F)	
prf.eccentricity	6.254	6.254	1	332.38	2.5116	0.1140	
hemifield	67.613	67.613	1	332.17	27.1549	3.305e-07	***
prf.eccentricity:hemifield	0.286	0.286	1	332.14	0.1150	0.7347	

DLPFC

	Sum Sq	Mean Sq	NumDF	DenDF	F value	Pr(>F)
prf.eccentricity	2.86748	2.86748	1	242.94	0.7709	0.3808
hemifield	0.62507	0.62507	1	243.34	0.1680	0.6822
prf.eccentricity:hemifield	0.98561	0.98561	1	243.12	0.2650	0.6072

Supplementary Note 7. PEGF center inversion

The horizontal component of the pEGF center (X_0) appeared to shift with pRF eccentricity from being contralateral to ipsilateral. We quantified the inversion point per ROI and visual field by estimating the linear relationship between pRF eccentricity and pEGF X_0. We computed two least-squares solutions, one for pRFs in the left, and one for pRFs in the right visual hemifield. Using the coefficients of these least-squares solutions, we computed where the two lines would intersect. If they did not intersect after 0 (i.e. in case of parallel or diverging lines), the intersection was set to 'NA'. We computed the median across the visual ROIs where we found an intersection point.

In addition to the pEGF parameter obtained with the optimal retinotopic representation of the visual input (i.e. where the contrast of the peripheral elements increased to 100% after saccade offset for 500 ms), we also computed the intersection after fitting the pEGFs using a retinotopic representation without any peripheral elements. In both cases the pEGF centers inverted from contralateral to ipsilateral (see Supplementary Figure 6B and S6C). The point of inversion was more central when the contrast of the peripheral elements increased to 100% (see table below). Based on the two different retinotopic representations, we expect the point of inversion to be approximately between 6 to 8 degrees from the fovea.

Supplementary Table 6

pRF eccentricity at which pEGFs become ipsilateral (°), i.e. point of inversion.

	**Contrast of peripheral elements**	
**ROI**	**100%**	**0%**
V1	6.5	8.4
V2	6.3	8.9
V3	5.9	7.8
V4	5.1	7.5
V3AB	5.8	7.2
IPS0-1	6.0	7.5
IPS2-3-4	NA	NA
LO1-2	9.8	18.6
TO1-2	3.2	5.0
Median	*5.9*	*7.9*
