# Supplementary material for: Topographic organization of eye-position dependent gain fields in human visual cortex
Source: Nat Commun. 2022 Dec 24;13:7925. doi: 10.1038/s41467-022-35488-8 (PMC9789150; doi:10.1038/s41467-022-35488-8)
Supplement: Supplementary file 3 — Description of Additional Supplementary Files [file 41467_2022_35488_MOESM3_ESM.pdf]

Description of Additional Supplementary Files

File name: Supplementary Movie 1

Description: The left panel displays the stimulus used for population receptive field (pRF) mapping. Participants were asked to fixate the central fixation point for an entire run. To the right of the stimulus, a retinotopic representation is displayed that we used to model the visual input during the pRF mapping paradigm.

File name: Supplementary Movie 2

Description: The left panel displays the stimulus used for version A of population eye-position dependent gain field (pEGF) mapping. Participants were asked to follow the fixation point with their eyes. To the right of the stimulus, a retinotopic representation is displayed that we used to model the visual input during the pRF mapping paradigm. We used different configurations of the model. In this example, a model is used where the peripheral visual elements act as a visual stimulus only after saccade offset, but not during fixation. Because the representation of the stimulus is retinotopic, the peripheral elements shift when the fixation point moves (and the participants have moved their eyes).

File name: Supplementary Movie 3

Description: The left panel displays the stimulus used for version B of population eye-position dependent gain field (pEGF) mapping. Participants were asked to follow the fixation point with their eyes. To the right of the stimulus, a retinotopic representation is displayed that we used to model the visual input during the pRF mapping paradigm. We used different configurations of the model. In this example, a model is used where the peripheral visual elements act as a visual stimulus only after saccade offset, but not during fixation. Because the representation of the stimulus is retinotopic, the peripheral elements shift when the fixation point moves (and the participants have moved their eyes).
