# Supplementary material for: Topographic organization of eye-position dependent gain fields in human visual cortex
Source: Nat Commun. 2022 Dec 24;13:7925. doi: 10.1038/s41467-022-35488-8 (PMC9789150; doi:10.1038/s41467-022-35488-8)
Supplement: Supplementary file 7 — Reporting Summary [file 41467_2022_35488_MOESM7_ESM.pdf]

nature portfolio

Corresponding author(s): J.H. Fabius, K. Moravkova, A. Fracasso

Last updated by author(s): May 4, 2022

Reporting Summary

Nature Portfolio wishes to improve the reproducibility of the work that we publish. This form provides structure for consistency and transparency in reporting. For further information on Nature Portfolio policies, see our Editorial Policies and the Editorial Policy Checklist.

Statistics

For all statistical analyses, confirm that the following items are present in the figure legend, table legend, main text, or Methods section.

n/a	Confirmed	
☐	☒	The exact sample size (*n*) for each experimental group/condition, given as a discrete number and unit of measurement
☐	☒	A statement on whether measurements were taken from distinct samples or whether the same sample was measured repeatedly
☐	☒	The statistical test(s) used AND whether they are one- or two-sided<br>*Only common tests should be described solely by name; describe more complex techniques in the Methods section.*
☐	☒	A description of all covariates tested
☐	☒	A description of any assumptions or corrections, such as tests of normality and adjustment for multiple comparisons
☐	☒	A full description of the statistical parameters including central tendency (e.g. means) or other basic estimates (e.g. regression coefficient) AND variation (e.g. standard deviation) or associated estimates of uncertainty (e.g. confidence intervals)
☐	☒	For null hypothesis testing, the test statistic (e.g. *F*, *t*, *r*) with confidence intervals, effect sizes, degrees of freedom and *P* value noted<br>*Give P values as exact values whenever suitable.*
☒	☐	For Bayesian analysis, information on the choice of priors and Markov chain Monte Carlo settings
☐	☒	For hierarchical and complex designs, identification of the appropriate level for tests and full reporting of outcomes
☐	☒	Estimates of effect sizes (e.g. Cohen's *d*, Pearson's *r*), indicating how they were calculated

Our web collection on statistics for biologists contains articles on many of the points above.

Software and code

Policy information about availability of computer code

Data collection	MATLAB 2019a The Mathworks Inc<br>Psychophysics Toolbox (Kleiner et al., 2007a)
Data analysis	R 3.6.3 (https://cran.r-project.org/)<br>lme4 package in R (https://cran.r-project.org/web/packages/lme4/index.html)<br>AFNI (19.2.10)<br>Python 2.7<br>Custom MRI segmentation and analysis toolbox https://github.com/AlessioPsych/AnalysisAfni<br>CRUISE algorithm implemented in nighres (https://github.com/nighres)

For manuscripts utilizing custom algorithms or software that are central to the research but not yet described in published literature, software must be made available to editors and reviewers. We strongly encourage code deposition in a community repository (e.g. GitHub). See the Nature Portfolio guidelines for submitting code & software for further information.

Data

Policy information about availability of data

All manuscripts must include a data availability statement. This statement should provide the following information, where applicable:

- Accession codes, unique identifiers, or web links for publicly available datasets
- A description of any restrictions on data availability
- For clinical datasets or third party data, please ensure that the statement adheres to our policy

Data to reproduce the figures in this in this study have been deposited in an OSF database (ADD LINK). The raw data contains individual-specific anatomical and

functional data, these are protected by current UK's implementation of the General Data Protection Regulation (GDPR). For this reason, the raw functional and anatomical data are available from the leading author upon reasonable request.

Field-specific reporting

Please select the one below that is the best fit for your research. If you are not sure, read the appropriate sections before making your selection.

☒ Life sciences ☐ Behavioural & social sciences ☐ Ecological, evolutionary & environmental sciences

For a reference copy of the document with all sections, see nature.com/documents/nr-reporting-summary-flat.pdf

Life sciences study design

All studies must disclose on these points even when the disclosure is negative.

Sample size	We aim at observing effects at the individual subject level. Thus, the number of subjects is needed to build confidence that the effect is reproducible across subjects, i.e. subjects are replication units not measurement units.<br>To get representative results in single subjects, we scan the subject more than once. Thus, we focus on sufficient trials per subject rather than sample size. For a discussion on the relationship and influence on statistical power between trials and sample size see Baker et al. (2021, DOI=10.1037/met0000337).<br>Typically, we aim for about 10 subjects. This is in line with many previous studies that use comparable experimental (model-based) approaches, for example Kay et al, Nature, 2008 (2 subjects, DOI: 10.1038/nature06713), Dumoulin and Wandell, Neuroimage, 2008 (6 subjects, DOI: 10.1016/j.neuroimage.2007.09.034), Huth et al, Nature, 2016 (7 subjects, DOI: 10.1038/nature17637), Harvey et al, Science, 2013 (8 subjects, DOI: 10.1126/science.1239052).
Data exclusions	No data were excluded
Replication	Cross-validation and modelling of the data under the null-hypothesis were performed
Randomization	All experimental tasks were performed by all participants. No randomization was required
Blinding	Blinding was not applicable, because all tasks were performed by all participants

Reporting for specific materials, systems and methods

We require information from authors about some types of materials, experimental systems and methods used in many studies. Here, indicate whether each material, system or method listed is relevant to your study. If you are not sure if a list item applies to your research, read the appropriate section before selecting a response.

Materials & experimental systems

n/a	Involved in the study	
☒	☐	Antibodies
☒	☐	Eukaryotic cell lines
☒	☐	Palaeontology and archaeology
☒	☐	Animals and other organisms
☐	☒	Human research participants
☒	☐	Clinical data
☒	☐	Dual use research of concern

Methods

n/a	Involved in the study	
☒	☐	ChIP-seq
☒	☐	Flow cytometry
☐	☒	MRI-based neuroimaging

Human research participants

Policy information about studies involving human research participants

Population characteristics	11 participants (3 authors), 7 female, 5 male, age range 24-37
Recruitment	Participants were recruited from the pool of potential participants managed and maintained by the University of Glasgow (Scotland), School of Psychology and Neuroscience. The experiment was advertised on a web-based interface. The pool complies with new 2018 General Data Protection Regulation policies.
Ethics oversight	Local ethics committee at the School of Medical, Veterinary and Life Sciences of the University of Glasgow (reference number: 200180191 and GN19NE455).

Note that full information on the approval of the study protocol must also be provided in the manuscript.

Magnetic resonance imaging

Experimental design

Design type	Event-related
Design specifications	Participants completed at least two scanning sessions, with one additional training session being completed before the first scanning session. Over the scanning sessions, participants completed at least 5 runs of pRF mapping (155 volumes, 310 seconds per run), at least 5 runs of eye movement task version A (179 volumes, 358 seconds per run), at least 4 runs of eye movement task version B (179 volumns, 358 seconds per run).
Behavioral performance measures	Eye tracking (median saccade latencies were 135 ms and 141 ms in the two eye movement tasks).

Acquisition

Imaging type(s)	functional, structural
Field strength	7T Siemens Magnetom Terra system
Sequence & imaging parameters	We collected T1-weighted MP2RAGE anatomical scans (anatomy) for each participant (0.625 mm isotropic, FOV = 160×225×240 mm3, 256 sagittal slices, TR = 4.68 ms, TE = 0.00209 ms, TI = 0.84 ms, TI1 = TI2 = 2.37, flip angle 1 = 5, flip angle 2= 6, bandwidth = 250Hz/px, acceleration factor = 3 in primary phase encoding direction).<br>During the tasks, functional data were acquired as T2*-weighted echo-planar images (EPI), using the CMRR MB (multi-band) sequence with the following acquisition parameters: resolution = 1.5 mm isotropic, FOV = 192×192×84 mm3, repetition time (TR) = 2000 ms, echo time (TE) = 50 ms, flip angle = 72°, multiband acceleration factor = 2, phase encoding direction = anterior to posterior. For the pRF mapping task we collected 155 volumes per run (310 s), and for the eye-movement tasks (versions A and B) 179 volumes (358 s). For each MRI session, we recorded 5 volumes with the same EPI sequence parameters with the phase-encoding direction inverted (posterior to anterior; top-up EPI) to correct for susceptibility-induced distortions and facilitate co-registration with the anatomical data. We acquired the top-up EPIs at the beginning of the MRI session (between the first and the second EPI run) and at the end of the MRI session (before the final EPI run).
Area of acquisition	Area of acquisition (field of view) covered known visual areas

Diffusion MRI ☐ Used ☒ Not used

Preprocessing

Preprocessing software

All analyses were performed in AFNI, R and Python (Nighres). Functional scans were slice timing corrected using the AFNI function 3dTshift.

For each MRI session, we computed a warp field to correct for geometric distortions from the original (non-motion corrected) EPI volumes, using the function 3dQwarp: we averaged the first 5 volumes of the EPI following the acquisition of the top-up EPI, and averaged the 5 volumes of each top-up EPI run. The resulting undistorted (warped) EPI volume is the halfway warping between the two average volumes.

EPI volumes from the active eye-movement task A and B were co-registered to the EPI volumes of the moving bar to obtain a voxel-by-voxel correspondence between tasks. The outcome of the coregistration for each participant and session was visually checked by evaluating the location of anatomical markers as gray matter/white matter (GM and WM, respectively) and GM/cerebro-spinal fluid (CSF) boundaries in the calcarine sulcus and the parietal cortex. All analyses on the functional data were performed on GM voxels in EPI space. For each voxel in the GM mask, we computed its normalized cortical depth, ranging from 0 (GM/WM surface to 1 (GM/cerebro-spinal fluid surface), based on a volumetric model. We excluded the inner and outer 10% of GM to exclude effects of partial volume. No spatial smoothing was applied.

The GM and WM were automatically segmented based on the anatomical scans using in-house software. Segmentation was performed on a downsampled resolution of the anatomical data (0.7 mm, isotropic, downsampling performed with the function 3dResample). T1-w images were co-registered to an atlas 60 to remove the cerebellum and subcortical structures. We separated the T1-w images in 6 different sections from posterior to anterior, with each section being used separately as an input for the 3dSeg function in AFNI to isolate the WM. The WM masks obtained from each part were summed together resulting in a whole brain WM mask. To derive the GM segmentation, we started from the obtained whole brain WM segmentation and an atlas co-registered to the T1-w images to acquire 35 regions per hemisphere. Next, a distance map from the WM/GM boundary to the pial surface was built computing the Euclidean distance of each voxel from the WM/GM border. Negative distances were assigned inside the WM and positive distances were assigned from the WM borders outwards. For each region, the coordinates were divided into four separate subparts using k-means clustering. This step was necessary to accurately delineate the boundaries on small portions of each region (subregion), with a highly homogeneous T1-w signal. For each subregion, voxels within -2 and 7 mm from the WM/GM border were selected and their T1-w intensity was stored for further analysis. For each region's subregion, we obtained 10 bins between -2 and 7 mm from the WM/GM border. For each bin, we computed the inter-quartile estimate of T1-w intensity. We calculated the 75% quantile of the inter-quartile estimates and computed the associated Euclidian distance from the WM/GM border. This Euclidean distance was taken as the cortical depth associated with the subregion. To improve the obtained GM segmentation, the WM and GM masks were fed to the "Cortical Reconstruction using Implicit Surface Evolution" (CRUISE) algorithm in Nighres.

Normalization | Data were not normalized to avoid distortions introduced by normalization

Normalization template | We aim at observing effects at the individual subject level, using a subject-specific ROI based approach. Thus, a normalization step was not necessary in our analysis pipeline.

Noise and artifact removal | Motion parameters between runs in a session were estimated by aligning the EPI volumes to the first volume of the first EPI run using the function 3dvolreg. To minimize interpolation applied to the EPI data, the motion estimates and warp field results were combined and applied in a single step, using the function 3dNwarpApply. Then, we computed the motion correction and warped mean EPI volume of the MRI session by averaging over all warped and motion corrected EPI volumes between runs and collapsed over all time points in the 'moving bar' paradigm. This resultant mean EPI volume was co-registered to the anatomy. First, we brought the anatomy and the session mean EPI volume into the same space by aligning their respective centers of mass. Next, the 'Nudge dataset' plugin in AFNI was used to manually provide a good starting point for the automated coregistration. This registration consisted of an affine transformation, using the local Pearson correlation as cost function in the function 3dAllineate. The individual motion-corrected runs were then de-spiked (using the function 3dDespike), scaled to obtain percentage BOLD signal change, and detrended with a 3rd order polynomial (using the function 3dDetrend). We averaged all processed EPI runs per task and participant to increase the signal to noise ratio. EPI time series were smoothed at the single voxel level with a 3-point Hamming window over time.

Volume censoring | Volume censoring was not applied as participants were extensively trained before data acquisition as were acquainted with the MRI environment. Secondly, we perform multiple acquisition of the same sequence and experiment, motion-correct, and averaged across acquisitions. Thus, minimizing the need for volume censoring.

Statistical modeling & inference

Model type and settings | Forward model (population receptive fields + extension described in the paper).

Effect(s) tested | Correspondence with predicted pRF time series: ordinary least-squares regression
Correlation between reconstructed and actual eye position: Pearson correlation
Relationship between pRF and pEGF parameters: linear mixed-effect model

Specify type of analysis: ☐ Whole brain ☒ ROI-based ☐ Both

Anatomical location(s) | Known visual areas; validated using pRF mapping

Statistic type for inference (See Eklund et al. 2016) | Voxel-wise

Correction | Bonferroni (where applicable)

Models & analysis

n/a	Involved in the study
☒	☐ Functional and/or effective connectivity
☒	☐ Graph analysis
☐	☒ Multivariate modeling or predictive analysis

Multivariate modeling and predictive analysis | Extension of population receptive field where gain of visual response is modulated by eye position and an eye-position dependent gain field.
